# An optimal proportion of mixing broad-leaved forest for enhancing the effective productivity of moso bamboo

**DOI:** 10.1002/ece3.1446

**Published:** 2015-03-17

**Authors:** Xiao-Fei Cheng, Pei-Jian Shi, Cang Hui, Fu-Sheng Wang, Guo-Hua Liu, Bai-Lian Li

**Affiliations:** 1 Collaborative Innovation Center of Sustainable Forestry in Southern China of Jiangsu Province, Bamboo Research Institute, Nanjing Forestry UniversityNanjing, 210037, China; 2 Centre for Invasion Biology, Department of Mathematical Sciences, Stellenbosch UniversityMatieland, 7602, South Africa; 3 Mathematical and Physical Biosciences, African Institute for Mathematical SciencesCape Town, 7945, South Africa; 4 Ecological Complexity and Modeling Laboratory, Department of Botany and Plant Sciences, University of CaliforniaRiverside, California, 92521-0124

**Keywords:** Biomass, environmental factors, generalized additive model, linear regression model, productivity

## Abstract

Moso bamboos (*Phyllostachys edulis*) are important forestry plants in southern China, with substantial roles to play in regional economic and ecological systems. Mixing broad-leaved forests and moso bamboos is a common management practice in China, and it is fundamental to elucidate the interactions between broad-leaved trees and moso bamboos for ensuring the sustainable provision of ecosystem services. We examine how the proportion of broad-leaved forest in a mixed managed zone, topology, and soil profile affects the effective productivity of moso bamboos (i.e., those with significant economic value), using linear regression and generalized additive models. Bamboo's diameter at breast height follows a Weibull distribution. The importance of these variables to bamboo productivity is, respectively, slope (25.9%), the proportion of broad-leaved forest (24.8%), elevation (23.3%), gravel content by volume (16.6%), slope location (8.3%), and soil layer thickness (1.2%). Highest productivity is found on the 25° slope, with a 600-m elevation, and 30% broad-leaved forest. As such, broad-leaved forest in the upper slope can have a strong influence on the effective productivity of moso bamboo, ranking only after slope and before elevation. These factors can be considered in future management practice.

## Introduction

The widely distributed moso bamboo (*Phyllostachys edulis*) is economically important in southern China for its palatable shoots and versatile culms. With its high growth rate, short rotation, high productivity, and early maturation (Zhou 1998), moso bamboo is an important forestry and economic plant in the bamboo production areas of southern China. The carbon stock of moso bamboo forest has been steadily increasing in recent decades, serving as a carbon sink in the subtropical region of China (Chen et al. 2009; Wang et al. 2013). Besides fixing carbon, moso bamboo forest can also provide other ecosystem services, such as water storage, soil proliferation, and biodiversity conservation (Hu et al. 2013), functioning as an important part in the regional forest ecosystem.

Mixing different plant species in managed habitats not only can increase the species diversity of other taxa, but also can potentially enhance the stability and productivity of the entire ecosystem (Tilman et al. 1996; Lehman and Tilman 2000; Shi et al. 2014a). Indeed, plant diversity has been shown to affect the water cycle in forest ecosystems by differentiating water consumption and recharging groundwater (Sprenger et al. 2013). Biodiversity may stabilize ecosystems simply by statistical averaging (Doak et al. 1998; Li et al. 2014) and enhance productivity under rather general conditions (Yachi and Loreau 1999). There are usually two patterns for a mixed forest: One is a discrete species mixture in a stand and another is a patchy mixture in a stand. For the former pattern, one species is sparsely distributed in the forest of another (or multiple) species, whereas for the latter pattern, two (or multiple) species exhibit mosaic spatial distributions. These two patterns are generally helpful for improving the plant productivity. However, the latter is easier to be handled in forest management.

Long-term intensive management can reduce the total and labile soil organic carbon stock in bamboo forests (Zhou et al. 2006; Li et al. 2013b). In particular, the soil fertility of pure bamboo forests gradually declines, threatening the sustainable productivity (Lou et al. 1997). In contrast, in mixed forests, broad-leaved trees normally have higher rates of soil nitrogen mineralization and nitrification, improving soil nitrogen concentration (Yan et al. 2008). Evergreen broad-leaved forest also holds soil moisture (Gong et al. 2011). All these functions of broad-leaved forest can make up for the deficiency of ecosystem service in pure bamboo forests. With these advantages of mixed forests, the tactic of mixing broad-leaved forest with moso bamboos by planting them in separate patches is frequently applied in the mountainous areas of southern China.

Concerning mainly about the productivity of moso bamboos, managers often deploy the strategy of mixing bamboos with broad-leaved trees. The Broad-Leaved forest In Upper Slope and Bamboo plantation In Lower Slope (BLIUSBILS) has become a common forestry management strategy in southern China (see Figs S1–S3 in the online Supporting Information). To date, few studies have yet explored the effect of upper slope broad-leaved forest on the lower slope bamboo forest. Here, we attempt to investigate the impact of the proportion of broad-leaved forest in a management unit on the bamboo productivity.

Vegetation productivity can be affected by many environmental factors (Liu et al. 2012) other than the aforementioned management strategy. For instance, bamboo density can also be a good indicator of management intensity (Zhou 1998). In mountainous regions, forest biomass and plant productivity are often closely related to elevation, slope, and slope aspect (Wang et al. 2006; Ming et al. 2011). Elevation has a complex influence on the biomass productivity and plant diversity in forests (Whittaker and Niering 1975; Whittaker 1978; Ermias et al. 2012), for example, through its influence on climate conditions such as temperature and precipitation (Blundo et al. 2012; Krömer et al. 2013; Li et al. 2013a; Alba et al. 2014). In the BLIUSBILS strategy, elevation and slope could affect the nutrient and water in moso bamboo forests flowed from broad-leaved trees.

These topographic variables can affect soil properties (e.g., soil organic matter content and concentration) (Dai and Huang 2006; Liu et al. 2012), soil nutrients (Wang et al. 2009), illumination intensity, and soil genesis (Jenny 1980), thereby driving the distribution pattern of plants (Fu et al. 2004; Sariyildiz et al. 2005). Specifically, plant establishment, growth, and distribution can be greatly influenced by soil factors (soil layer thickness, humus layer thickness and rock content) (Liu et al. 2012).

To investigate the impact of the proportion of broad-leaved forest, as well as other factors, on moso bamboo productivity, we here investigate 80 mountains with different proportions of broad-leaved forest. We use linear regression and generalized additive models for analyzing the relationship between moso bamboo forest productivity and the proportion of broad-leaved forest, together with density, elevation, slope, slope location, slope aspect, soil layer thickness, gravel content, and humus layer thickness as explanatory variables. We identify the dominant factors of each variable by calculating its contribution rate using the Akaike information criteria (AIC; Akaike 1974). We also discuss the optimum value of each variable for maximum productivity of moso bamboos. The results provide important theoretical basis for forestry management in the region.

## Materials and Methods

### Experimental design

**Figure 1:**
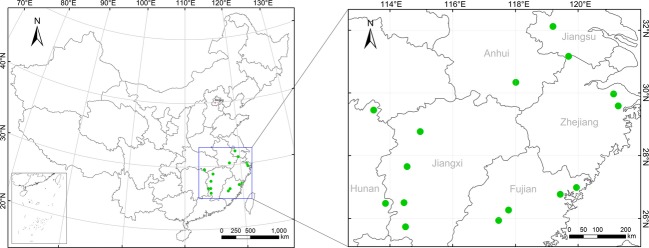
The distribution of sampling locations.

The study area extends across six provinces in southern China, including Fujian, Hunan, Jiangxi, Zhejiang, Anhui, and Jiangsu provinces. We selected 2–4 locations in each province that are well known for bamboo production. In each location, we selected four mountains that are growing moso bamboos in the scope of the BLIUSBILS management strategy. In total, 15 locations (Fig.1) and 80 mountains in six provinces were selected, stretching between 25°44′-32°08′ N and 113°24′-121°18′ E, from 60 to 1500 m in elevation.

Field experiments were conducted from October 2013 to June 2014. We surveyed each mountain by four transects: 1 in the broad-leaved forest and 3 evenly distributed in the bamboo forest across the elevation range. Each transect includes three 20 × 20-m^2^ plots. A total of 960 plots were surveyed. In the broad-leaved tree plot, all trees were identified, with their diameters at breast height measured and tree height estimated. In the bamboo plot, the diameters at breast height and the total number of bamboos were recorded. Then the density of moso bamboo each plot was calculated by using the total number of bamboos in the plot divided by the corresponding plot's area.

The proportion of broad-leaved forest in a mountain was estimated on a map for the area stretching the entire slope. Topographic factors were also recorded for each mountain, including elevation, slope, slope aspect, and slope location (upper level, middle level, and lower level). Soil factors in each plot were obtained by digging a soil profile (1.5–2.0 m in length and 1 m in width; the depth depends on different soil types), with the humus layer thickness, soil layer thickness, and gravel content recorded.

### Statistical analyses

**Figure 2:**
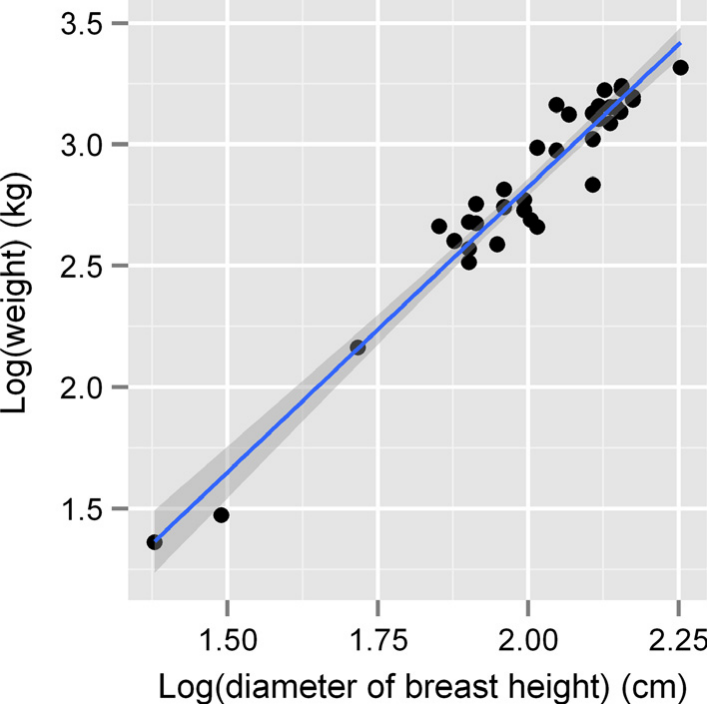
The relationship between the diameter at breast height (DBH) and the biomass of moso bamboo. The data of 36 bamboos (DBH > 10 cm) were collected in Xiashu Forest Station, Zhenjiang City, Jiangsu Province (32°07′58.3″N, 119°12′08.5″E) in the early June, 2014.

We consider biomass as a measurement of productivity. In general, dry weight and wet weight are often used as the indicators of plant biomass, yet in practice they are strongly correlated (Shi et al. 2013). Moreover, weight can be further estimated from the length or height of the focal species by an allometric relationship as previously demonstrated (Makarieva et al. 2004; Shi et al. 2013, 2014b). As it is impossible to cut all bamboos for measuring the biomass and weight, we used a strong allometric relationship between biomass and diameter at breast height (DBH) (Zhou 1998), parameterized using 36 moso bamboos (Fig.2). DBH can represent biomass very well. However, there are individual differences in DBH among moso bamboos in any a plot (Sandhu et al. 2013). Consequently, we used the average DBH in a plot as a proxy of bamboo biomass. In practice, moso bamboos with DBH ≥ 10 cm have significant economic value. And these individuals also have large individual biomass as the DBH and weight have a strong allometric relationship (Fig.2). Below, we will focus on effective productivity of moso bamboo using the average DBH each plot, rather than stand productivity using the total biomass each plot. There are probably many individuals in a plot, which means high stand productivity. However, the average DBH of moso bamboo might be very small, which leads to low effective productivity. Thus, using the average DBH appears to be more reasonable for representing effective productivity of moso bamboo relative to using the total biomass of moso bamboo in a plot.

Data analysis was performed through R 3.0.0 (R Development Core Team 2013). We first conducted multiple linear regressions of the density of moso bamboo (as a factor reflecting the influence of the density dependence on individual biomass) and eight environmental factors (the proportion of broad-leaved forest, elevation, slope, slope aspect, slope location, soil layer thickness, humus layer thickness and gravel content) on the average DBH of moso bamboos in plots. Second, because there may exist nonlinear relationships between environmental factors and the DBH, we used the generalized additive model (GAM) (Hastie and Tibshirani 1990; Wood 2006).

To increase the prediction power of the regression equation, variables with little relevance to the dependent variable (DBHs) were removed according to the value of AIC. We then obtained an AIC score (AIC_0_) for using all six remaining environmental factors as independent variables in the GAM. By removing one specific independent variable, we obtained an increased AIC score (AIC_*i*_) due to the removal. The contribution rate (CR_*i*_) of each independent variable can be calculated by the following equation:

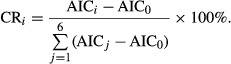


Because the slope location is a categorical variable, we ran a Shapiro–Wilk test (Xue and Chen 2007) for examining the relationship between slope location and the DBHs of moso bamboos. When the DBHs of different slope locations did not follow a normal distribution, we ran a Kruskal–Wallis test (Xue and Chen 2007) instead. We also drew the partial residual plot (Hastie and Tibshirani 1990; Wood 2006) for the primary variables affecting the DBHs of moso bamboo and identified values of these variables at which the highest productivity of moso bamboos was achieved.

## Results

The ranges of all measured variables in this study are presented in Table1. The multiple linear regression model (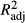 = 0.092, *P* < 0.01) for all nine variables performed much worse than the GAM (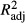 = 0.313). Consequently, the GAM was used for subsequent analyses. According to the AIC, the GAM performed the best when six variables were used (Table2), including the proportion of broad-leaved forest, elevation, slope, slope location, soil layer thickness, and gravel content. The GAM for these six variables (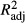 = 0.333) performed much significantly better than the linear regression model (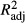 = 0.068, *P* < 0.01). In particular, of these six variables, we found no significant influence of soil layer thickness on the DBH of moso bamboo (*P *>* *0.05) (Table2). In contrast, the proportion of broad-leaved forest, elevation, slope, and gravel content had significant impacts on the DBHs (Table2).

**Table 1:** A description of variables and their ranges in the study

Variable	Mean	Standard error	Minimum	Maximum
DBH each individual (cm)	10.0	2.0	3.5	16.7
Average DBH each plot (cm)	10.1	1.4	6.3	14.6
Density (per ha)	2929	1066	900	6600
Proportion of broad-leaved forest (%)	22.3	16.4	0.0	70.0
Elevation (m)	646.5	406.2	66.3	1250.0
Slope (°)	30.3	8.3	3.0	49.0
Gravel content by volume (%)	19.5	16.8	0.0	82.0

**Table 2:** Results from the generalized additive model (GAM) in explaining the DBH of moso bamboos (*n* = 238)^1^

Independent variable	Estimate	*t* Value	Pr (>|*t*|)	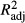
Intercept	10.019	53.934	< 0.01	0.33
Slope location (lower slope)	0.572	2.871	< 0.01	
Slope location (middle slope)	0.333	1.805	0.073	
Soil layer thickness	−0.341	−1.814	0.071	
	df	*F* Value	*P* value	
s(elevation)	8.951	3.557	<0.001	
s(broad-leaved proportion)	8.306	3.328	0.001	
s(slope)	8.541	3.409	<0.001	
s(gravel content by volume)	8.782	2.608	0.008	

1 In this table, s(·) represents the smooth function.

**Figure 3:**
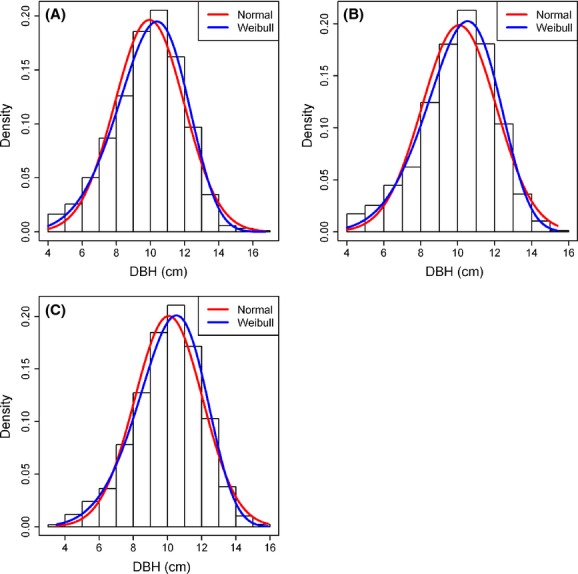
The distribution of DBH for moso bamboos at different slope locations: (A) upper level, (B) middle level, and (C) lower level.

**Figure 4:**
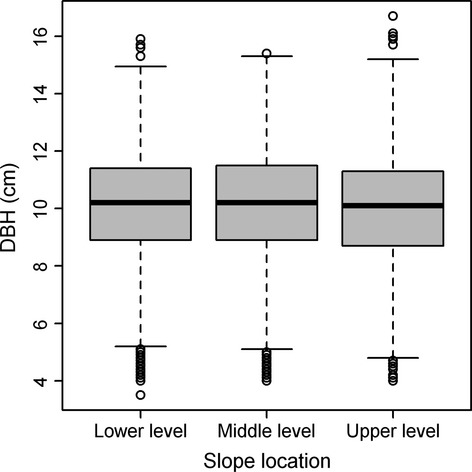
Box plots of moso bamboo DBHs in different slope locations.

The Shapiro–Wilk test showed that the DBH at different slope locations did not conform to a normal distribution, but followed a Weibull distribution (Fig.3). After running a Kruskal–Wallis test, we detected a significant difference in DBHs at different slope locations (*χ*^2^ = 7.9, *P *=* *0.02) (Fig.4), consistent with the results from the GAM (Table2).

**Figure 5:**
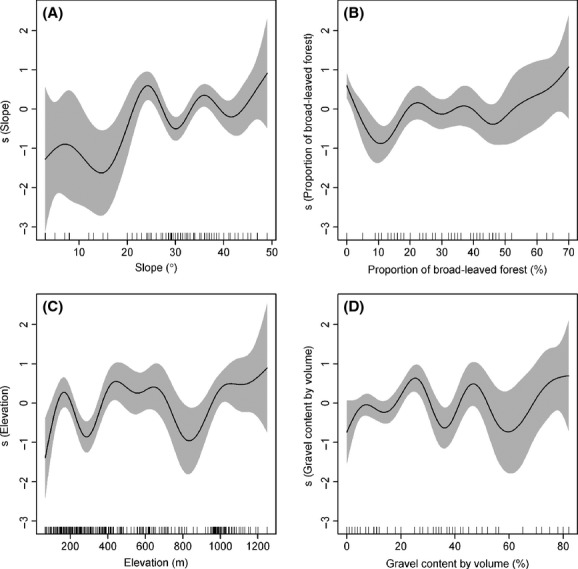
Partial residual plots of the top four explanatory factors.

The contribution rates (CR) of these six variables to bamboo productivity (i.e., the DBH) are, in order, slope (25.9%), the proportion of broad-leaved forest (24.8%), elevation (23.3%), gravel content by volume (16.6%), slope location (8.3%), and soil layer thickness (1.2%). Notably, we confirmed the significant impact of broad-leaved forest on bamboo productivity in the scope of the BLIUSBILS management strategy. In addition, the slope of 25° (Fig.5A), 30% proportion of broad-leaved forest (Fig.5B), and the elevation of 600 m are helpful for improving the productivity of moso bamboo (Fig.5C).

## Discussion

Our results suggest that the four most important factors of concern in the BLIUSBILS strategy for maximizing bamboo production are slope, the proportion of broad-leaved forest, elevation, and gravel content. First, the slope of the terrain drives the spatial distribution of soil nutrients and water availability (Fisher and Binkley 2000; Costa et al. 2008; Wang et al. 2009) which can have a great impact on plant communities (Weltzin et al. 2003). Different amount and seasonality of water availability could cause the fluctuation and decline of plant productivity (Swemmer et al. 2007; Wu et al. 2011). Mountains with steep slopes or convex slopes have a high erosion risk and thus negatively affect forestry productivity (Wadaey and Ziada 2014). Second, the additional broad-leaved forest can provide a better supply of soil water and soil nutrient to affiliated bamboo forests (Yan et al. 2008). This is because soil respiration rate in broad-leaved forests is higher than in other forest types (Wang et al. 2004). For instance, converting coniferous forests into broad-leaved forests can increase water yield (Komatsu et al. 2008). As such, the upper slope broad-leaved forest can have a positive impact on the lower slope bamboos. Finally, elevation plays an important role in plant productivity because with the increase of elevation, air temperature normally drops and the precipitation increases (Körner 2003). Such changes in temperature and precipitation can affect ecosystem functioning (Cramer et al. 2010), such as global primary production (Zhao and Running 2010) and plant species richness (Krömer et al. 2013; Carlyle et al. 2014) in different ways. Elevation can also affect soil nutrients, such as soil organic matter (Dai and Huang 2006), and total N, P, and K (Liu et al. 2012), which are needed for plant establishment and growth.

In our study, 74% variance of the DBH of moso bamboos was jointly explained by slope, the proportion of broad-leaved forest, and elevation. As slope and elevation are topographic factors that cannot be modified, controlling the proportion of broad-leaved forest, by planting or removing broad-leaved trees on the upper slope, becomes an essential component for successfully implementing the BLIUSBILS management strategy.

Bamboo production reached the peak at the slope of 25°, in line with the observation that durian yields are greater on gentle and moderate slopes than on flat and steep slopes (Salafsky 1995). Indeed, steep slope has been found having a negative effect on cork production, tree size (circumference at breast height), and tree density (Costa et al. 2008). In the scope of the BLIUSBILS management strategy, a certain level of slope is beneficial for increasing sunlight intensity received by bamboo forests. Slope also affects the water and nutrient absorption by moso bamboos provided by the upper slope broad-leaved forest. When the slope is too steep, water and nutrients from the broad-leaved forest will flow quickly, reducing the amount of water and nutrient can be captured by bamboos. In contrast, with gentle slopes, little water and nutrients will be transported from the broad-leaved forest to the bamboo forest.

The DBH of moso bamboo reaches the peak when the upper slope broad-leaved forest covers 30% (ranging 20% to 40%) of the total slope area, suggesting an optimal proportion of broad-leaved forest for soil nutrient cycling and water conservation in moso bamboo forests. If the proportion of broad-leaved forest is too high, the broad-leaved trees become dominant and could negatively affect the bamboo growth. If the proportion is too low, the water and nutrient supply from the broad-leaved forest might not be sufficient for the bamboo forest. A further investigation could be to examine how species composition, tree age, and size of broad-leaved forest affect bamboo productivity.

The DBH of moso bamboo varies with elevation and peaks at the elevation of 600 m (ranging from 500 to 700 m), consistent with the management advice for high-yield bamboo forests in southern China (Zhou 1998): an elevation above 600 m on a sunny slope <30° for moso bamboos. This advice meets the need of moso bamboo as being both thermophilic and hygrophilous. Bamboo forests at elevation too high or too low suffer from harsh climate conditions of extreme temperature, strong wind, and excessive rainfall (Chen et al. 1992).

In addition, slope location, affecting sunshine duration and the degree of solar radiation, has demonstrated a significant effect on the height and coverage of shrubs and herbs (Liu et al. 2011). Results from the GAM did suggest that slope location presents a substantial impact on the DBH of moso bamboo. The Weibull distribution of bamboo DBH, detected here, could serve as a reference for future studies. Moreover, as the source of heat energy, temperature, especially the accumulated temperature, plays an important role in plant growth and productivity (Olivier and Annandale 1998; Sacks and Kucharik 2011). We did not consider this variable here due to difficulties in obtaining the micro-meteorological data for the 15 locations. However, the effect of effective temperatures has been, to a certain degree, represented by considering the elevation in our study given their close relation (Körner 2003). In mountainous areas, climatic conditions are closely related to the variation of elevation (Rahbek 2005). This could compensate the lack of climatic variables in our analysis.

In conclusion, in the scope of the BLIUSBILS management strategy, the proportion of broad-leaved forest on the upper slope has a great influence on the bamboo productivity, with a contribution rate of 24.8%, just after slope (25.9%), and before elevation (23.3%). Besides these three factors, the productivity of moso bamboo is largely determined by other topographic factors (slope location) and soil factors (gravel content and soil layer thickness). For maximum productivity, the optimum values of slope, the proportion of broad-leaved forest, and elevation are, respectively, 25°, 30%, and 600 m. The DBH of moso bamboo does not follow a normal distribution, but a Weibull distribution. As the slope and elevation are topographic factors, we can only manage the proportion of upper slope broad-leaved forest to enhance bamboo productivity. Further analysis on the effect of the community structure of the broad-leaved forest on bamboo productivity warrants attention in the BLIUSBILS management planning.

## Supporting Information

**Figure S1.** Illustration of the Broad-Leaved forest In Upper Slope and Bamboo plantation In Lower Slope (BLIUSBILS) management pattern. **Figure S2.** Broad-leaved forest on the peak of a mountain, Yongan City, Fujian Province, China. **Figure S3.** Moso bamboo forest in a mountainous area of Yongan City, Fujian Province, China.
