# Supplementary figures and images for: An optimal proportion of mixing broad-leaved forest for enhancing the effective productivity of moso bamboo
Source: Ecol Evol. 2015 Mar 17;5(8):1576–84. doi: 10.1002/ece3.1446 (PMC4409407; doi:10.1002/ece3.1446)

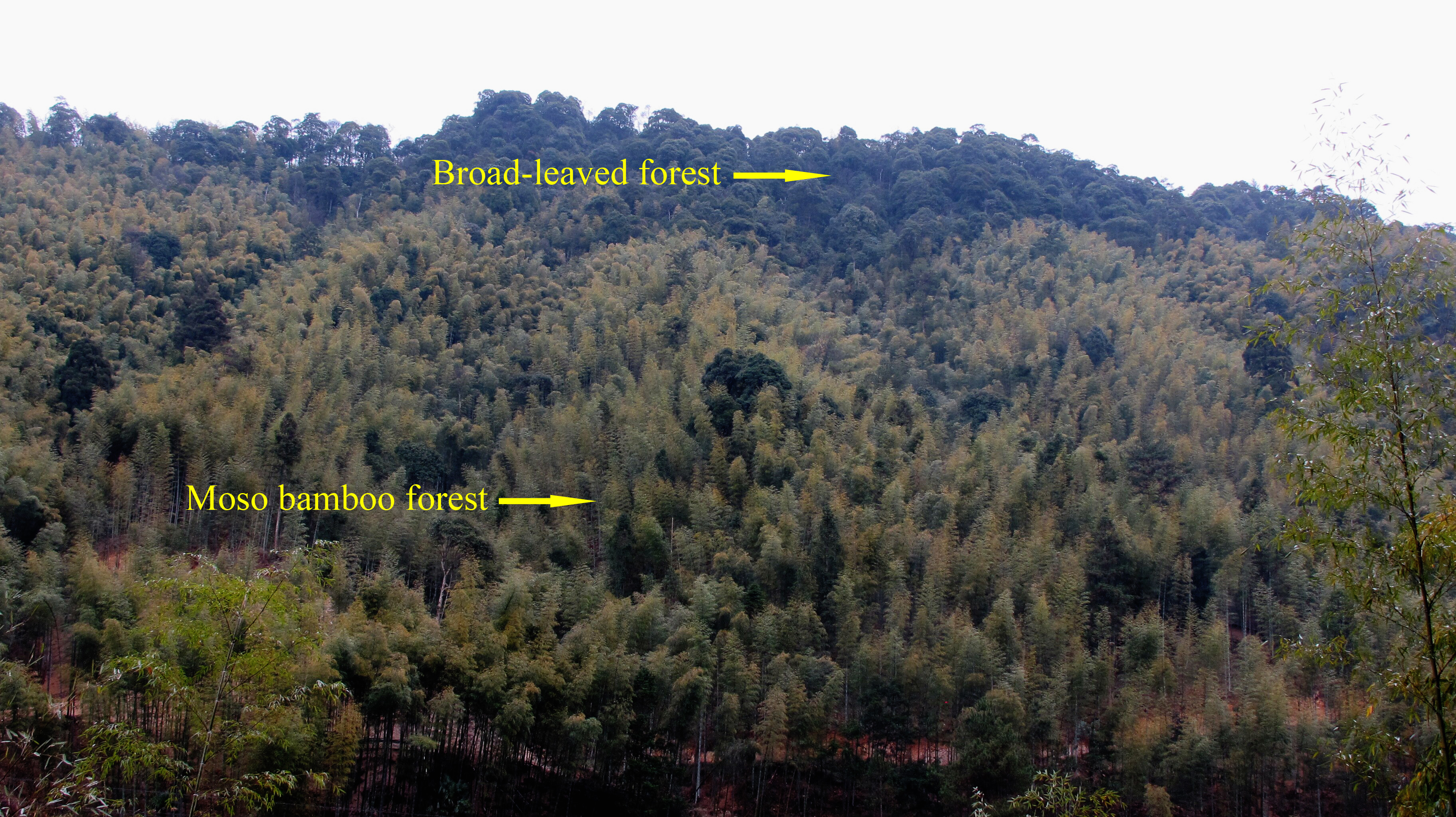

Supplement: Supplementary file 1 [file ece30005-1576-sd1.zip › ECE3_1446_S1.jpg]
