# Supplementary material for: An optimal proportion of mixing broad-leaved forest for enhancing the effective productivity of moso bamboo
Source: Ecol Evol. 2015 Mar 17;5(8):1576–84. doi: 10.1002/ece3.1446 (PMC4409407; doi:10.1002/ece3.1446)
Supplement: Supplementary file 1 [file ece30005-1576-sd1.zip › Supporting Information.doc]

Supporting Information:

**The case of broad-leaved forest in upper slope and bamboo plantation in lower slope (BLIUSBILS) in southern China**

**Figure S1.** Illustration of the Broad-Leaved forest In Upper Slope and Bamboo plantation In Lower Slope (BLIUSBILS) management pattern. The dark green part represents broad-leaved trees, and the light green part represents moso bamboos. This photo was took in a mountainous area (25°59'57.7'' N, 117°31'56.9'' E) of Yongan City, Fujian Province, China

**Figure S2.** Broad-leaved forest on the peak of a mountain, Yongan City, Fujian Province, China

**Figure S3.** Moso bamboo forest in a mountainous area of Yongan City, Fujian Province, China
